# Integrated operations for natural disaster management: A systematic Review

**DOI:** 10.34172/hpp.2022.33

**Published:** 2022-12-10

**Authors:** Yousef Pashaei Asl, Mohsen Dowlati, Javad Babaie, Hesam Seyedin

**Affiliations:** ^1^Department of Disaster & Emergency Health, School of Health Management & Information Sciences, Iran University of Medical Sciences, Tehran, Iran; ^2^Health Management and Economics Research Center, Health Management Research Institute, Iran University of Medical Sciences, Tehran, Iran; ^3^Road Traffic Injury Research Center, Department of Health Policy and Management, School of Management and Medical Informatics, Tabriz University of Medical Sciences, Tabriz, Iran; ^4^Department of Health Policy & Management, Tabriz University of Medical Sciences, Tabriz, Iran

**Keywords:** Natural disaster, Logistics, Model

## Abstract

**Background:** This study aimed to conduct a systematic review of models describing the integrated logistics operations performed as a response to natural disasters, with the hope to identify the challenges and limitations of healthcare systems in natural disaster management.

**Methods:** A systematic literature search was carried out in PubMed/Medline, Scopus, Google Scholar, and bibliographies of retrieved articles using MeSH headings and keywords such as natural disaster, logistics, model. A total of 98 publications were identified through the search process. Seven potentially relevant articles met the inclusion criteria. The key demographic, clinical, and pathological information of all qualified studies were extracted from the full-text articles.

**Results:** Among the seven included studies, six had either model data or considerations on distribution methods. Storage, human resources, infrastructures, primary priority items, coordination of organizations, and information and communication with the media were also the focus of studies. The articles were mainly from Iran (n=2), the United States (n=2), and Indonesia (n=2). The models presented in the studies has mainly focused on a specific aspect of disaster management, such as smart government development, use of military services, people with logistic training and/or medical team model.

**Conclusion:** This study systematically highlighted the crucial points that should be considered in managing natural disasters including human resources, infrastructure, storage, priority items, distribution, access system, coordination of organizations, information, and communication with the media. In this regard, we prepared a comprehensive comparison of possible models and logistics.

## Introduction

 Natural disasters cause significant casualties and economic damage. According to the United Nations Office for Disaster Risk Reduction (UNISDR 2019), a disaster is “a serious disruption of the functioning of a community or society at any scale caused by hazardous events interacting with conditions of exposure, vulnerability, and capacity, resulting in one or more of the following: human, material, economic, and environmental losses and impacts”.^1^ Earthquakes, tsunamis, volcanic eruptions, floods, hurricanes, and disease outbreaks are all instances of disasters. These disasters put people in perilous situations, and necessitate immediate medical attention.^2,3^

 The importance of emergency management is evident in today’s world. Most of human tragedies and infrastructure devastations can be prevented with forethoughtful and specific planning. No country and/or town is immune to the threat of natural disasters. However, societies may be prepared for effective responding to, and recovering from disasters, in a way that could limit any possible destruction to a certain extent. The discipline of preparing for and coping with dangers is known as emergency management (or disaster management).^4^ As a never-ending process, emergency management is a field of study that entails preparing for disasters before they occur, responding to disasters as soon as they occur, and sustaining and rebuilding societies after natural and/or human-made disasters strike. Based on emergency management, it is critical to have complete emergency action plans in place, and regularly analyze and enhance such plans. Preparedness, Response, Recovery, and Mitigation are the four phases of the connected activities in the action plans. Appropriate actions at each stage of this cycle may result in higher preparedness, better warnings, reduced susceptibility, and disaster prevention during the next cycle iteration.^5^

 During calamities, numerous relief organizations frequently encounter serious challenges in moving extensive amounts of various commodities such as food, clothing, medicine, medical supplies, machinery, and also manpower from various points of origin to diverse destinations in disaster areas. Supplies and relief personnel must be transported promptly and efficiently to optimize the survival probability of the afflicted people while minimizing the expense of such activities.

 This research aims to conduct a comprehensive systematic review on previously published models, describing integrated logistics operations in response to natural disasters, and identifying challenges and limitations of healthcare systems in terms of natural disaster management.

## Material and Methods

###  Search strategy

 A comprehensive literature search was carried out in PubMed/Medline, Scopus, Google Scholar, and bibliographies of retrieved articles. Searches were undertaken using MeSH headings and keywords, including Health needs, health kits, health pack, health items, relief kits, food supply, logistics, supply chain, disaster, earthquake, emergencies, disaster and catastrophe. The search period covered all years from the inception to April 2022.

###  Inclusion criteria

 The inclusion criteria were as follows: (1) qualitative and quantitative studies eligible up to April 2022; (2) articles with available data about logistic models in natural disaster management. The studies meeting the following criteria were excluded: (1) Original articles not containing data on logistic models for the management of natural disasters; (2) Articles that were inaccessible after two times of contacting the corresponding author; (3) Articles with unclear data description. Two reviewers independently examined the articles for inclusion. For studies reported in more than one publication, data abstraction was performed using all publications; however, one report was only included.

###  Study selection process

**Figure 1:**
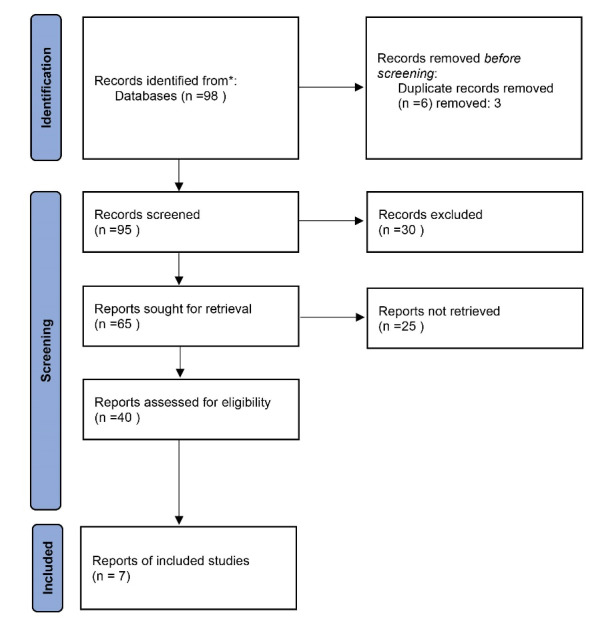
The diagram of the study based on PRISMA checklist

 The study selection process began with a title and abstract screening by two independent reviewers (HS and YP). The selection was based on the inclusion criteria. Articles passing the initial screen were then retrieved and reviewed by HS and YP. A third author resolved any disagreements between the two reviewers during the search and study selection process. Duplicate studies were excluded. The diagram of the study based on PRISMA checklist is presented in Figure 1.

 The information was gleaned from full-text articles. The data extraction was carried out separately by two authors (HS and YP) using the population, intervention, control, and outcomes (PICO) principle.

###  Outcome measures

 This review aimed to systematically identify and summarize all published articles concerning supply chain and logistics in natural disaster management, including human resources, infrastructure, storage, primary priority items, distribution and access system, coordination of organizations, and information and communication with the media.

###  Data extraction

**Figure 2:**
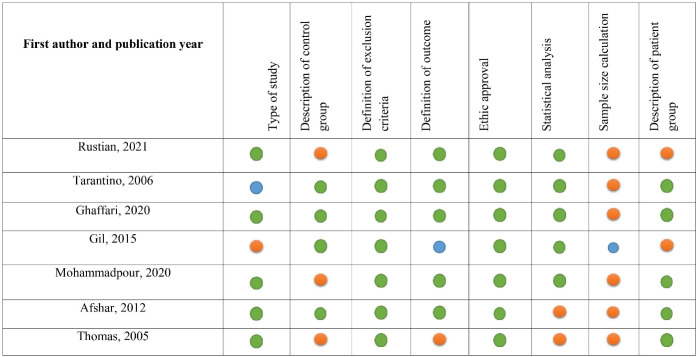
Quality control and risk of bias of included studies

 Data were extracted from full-text articles. Table 1 summarizes the essential demographic, clinical, and pathological information from all qualifying data sources. These data contain the initial author’s name, year, country, human resources, infrastructure, storage, priority items, distribution, access system, organizational coordination, information, and media communication. In the cases where vital information was missing from the text, the authors of the articles were contacted. The methodological quality was judged using the STROBE statement check list.^6^ The qualitative review items are presented in Figure 2.

**Table 1:** The main characteristics of the included studies and their information

**Author**	**Year**	**Country**	**Human resources**	**Infrastructure**	**Storage**	**Primary priority items**	**Distribution and access system**	**Coordination of organizations**	**Information and communication with the media**
Wardhono ^7^	2021	Indonesia	Human resource areas have no support and regulations are unclear.	Each region is given an e-logpal account to monitor equipment and logistics data.	-	-	Global access to a summary of available equipment through applications on communication devices such as Android mobile phones	Inefficiency of policies of the government officials in the management of natural disasters in Indonesia and the proposal to regulate better in the comprehensive management of disasters	Support and willingness to implement smart government due to public use of electronic means of communication
Tarantino^8^	2006	Sri Lanka & Indonesia	Military and civilian participation	DoD used sea and air routes to deliver the required items.	Relocation of emergency supplies from US civilian and military depots and initial allocation of emergency aid to affected countries	- Basic medical equipment for the treatment of trauma and acute illness - Drinking water sanitation - Facilities to prevent the spread of the disease - Infectious disease monitoring systems	Use of sea and air routes for delivery of vital medical equipment and drinking water supply by the DoD	DoD worked with WHO to ensure deployment of a senior health action in crisis	Gather US government stakeholders via teleconferences and meetings to develop coordinated strategy for health sector assistance, international coordination, and media outreach.
Ghaffari^9^	2020	Iran	The medical team model as a renewable resource.	Used the particle swarm optimization algorithm to solve the problem on a small and large scale.	The network includes hospitals where injured people receive emergency assistance.	Consider and supply two types of rescue kits (standard and custom) containing medical items as non-renewable resources	Given the lack of sufficient vehicles to carry relief items to deliver items to multiple destinations, considering routing decisions for both sources can be more complex.	-	- Formal interviews (in person, telephone or video conference) to refine the process of identifying secondary sources and the role of actors in food aid the distribution - Collection of all articles and media reports for the initial identification of actors in the distribution of food aid
Gil^10^	2015	Colombia	-	-	- UN Cluster Approach: Import food aid goods from the UN Humanitarian Warehouse in Panama - UARIV: Relying on domestic private food markets when starting food aid deployment	Food aid packages	- UN Cluster Approach: Leaving warehousing and transportation to 3^rd^ party logistics - UARIV: Delivery of food aid kits to local authorities and material handling and downstream transportation management by them	-	-
Mohammadpour^11^	2020	Iran	-Hospital staff in the early hours -Social mental health team	-	Disposable items such as stents, balloons, IUDs, and pacemakers were stored as strategic items.	Toothpaste, toothbrush, sanitary pad, mother-child packs	-Assisting various companies in the province in earthquakes - Setting up a field hospital and a mobile medical system - Existence of land transportation system and absence of air transportation system	Coordination between governmental and non-governmental organizations and cooperation with stakeholders and sharing of resources such as equipment as well as intergovernmental and non-governmental experiences	-
Afshar^5^	2012	USA	-	-Four potential locations for Federal Mobilization Centers - Four potential locations for Federal Operations Areas - Ten potential sites for the State Staging Areas	A commercial storage site in North Carolina, and a vendor in Nashville are used to store relief items.	Water (beverages and non-potable), meals, portable shelter, basic medical kit, bed, blanket, ice, baby supplies, generator, tunic, plywood, nail	Use only one mode of transport (cargo): One hundred 53-foot trailer trucks with a volume capacity of approximately 6000 feet each	Expansion of the complex supply chain across the country to coordinate with its government and local counterparts and with non-profit and for-profit organizations	-
Thomas^12^	2005	USA	- Assessment team to determine the needs of the population - Logistics team - People who can manage a complex relief supply chain - Warehouse providers	Use of people with logistic training	-	-	-	-	- Use of tracking and manual tracking software - Extensive use of Excel spreadsheets or manual processes to update and track goods entering the field

UARIV: Unit for Assistance and Reparation of Victims approach; UN: United Nations; DoD: Department of Defense; US: United States; IUDs, intra-uterine devices.

## Results

###  Study characteristics

 As shown in Figure 1, 98 publications were identified through database searching and other sources. After removing duplicates (three studies), 95 studies were included for initial screening. Based on the exclusion criteria, 55 articles were excluded in the first screening, due to the reasons like having unrelated purposes and unclear indicators. Abstracts of 40 potentially related studies were obtained (Figure 1). Subsequently, the full-text of articles were reviewed carefully, within which 33 studies were omitted because they did not analyze supply chain and logistics, and/or report their associated results. Full-text articles of seven potentially relevant citations met the inclusion criteria and were included for review. The main characteristics of included studies and their information are summarized in Table 1.

###  Main findings

 The main characteristics and outcomes of included studies and their associated model information are summarized in Table 1. Among the seven included studies, six had model data and/or considerations on distribution methods. Storage (five studies), human resources (five studies), infrastructures (five studies), primary priority items (five studies), coordination of organizations (four studies), and information and communication with the media (four studies) were also the focus of studies.

 Ghaffari et al studied a supply chain network that includes local and global medical relief item providers, regional and central distribution facilities, and several client demand points.^9^ This network included hospitals where injured people receive emergency assistance. Furthermore, in terms of primary priority items, a capacitated multi-stage operations scheduling problem was provided, in which renewable (medical teams) and non-renewable (standard and/or customized medical kits) resources were required in the emergency supply chain. A new mixed integer programming paradigm was also given and resolved for small-scale problems. A particle swarm optimization approach was also developed to solve large-scale challenges. Finally, the performance of the proposed solution method was evaluated, and some sensitivity assessments on the main significant parameters were performed. This study also suggested the use of formal interviews (in person, telephone and/or video conference) to refine the process of identifying secondary sources, and the role of actors in food aid distribution, and the collection of all articles and media reports for the initial identification of actors in the distribution of food aid.

 In another study, Wardhono applied public policy theory, smart governance, and supply chain management and logistics.^7^ The outcomes of their study were founded on ontology, epistemology, and sociology. They achieved smart governance through empowering supply chain and logistics to better catastrophe management. This study, for the first time, suggested a logistic plan, in which each region was given an e-logpal account to monitor equipment and logistics data, and global access to a summary of available equipment, through applications on communication devices, such as Android mobile phones.

 Afshar and Haghani also devised a mathematical model that regulated the movement of different relief goods from their origins, through a supply chain, into the hands of beneficiaries.^5^ Their suggested model took into account not only vehicle routing and pick-up/delivery schedules, but also the best locations for numerous layers of temporary facilities, as well as several capacity limits for each facility and a transportation system. A series of numerical experiments was also constructed to test the suggested formulation, and to evaluate the optimization problem’s attributes. Eventually, the study introduced four potential locations for Federal Mobilization Centers, four potential locations for Federal Operations Areas, ten potential sites for the State Staging Areas, and only one form of transportation (cargo) to be used for distribution and access system. This numerical study demonstrated the model’s ability to handle large-scale relief operations in sufficient detail. However, by extending the challenge, the size and difficulty of problems might be dramatically arisen.

 Tarantino investigated some fundamental policy concerns and strategic coordination and planning methods applied in the public health responses to the December 26, 2004 Asian tsunami.^8^ However, due to the significant level of heterogeneity among the introduced models in the associated studies, a meta-analysis was not possible. The Asian tsunami relief efforts demonstrated the need of civil-military collaboration in disaster relief, particularly in public health. The Department of Defense’s (DoD) major engagement in tsunami relief operations, particularly public health initiatives, had positive secondary effects through strengthening security cooperations, and gaining “hearts and minds” in the region. They used air and sea routes, through available resources by DOD, to deliver the prioritized items, including vital medical equipment and drinking water supply. They also worked with WHO to ensure deployment of a senior health action in crisis.

 Utilizing substance examination method, Mohammadpour et al conducted a subjective study on the Iranian’s healthcare framework challenges amid to characteristic calamities.^11^ Beside the health sector challenges with either intersectoral or intrasectoral nature, the non-health sector challenges were mostly social and mental challenges, which may be considered as the major challenges of Iran’s healthcare framework while confronting emergencies. In this respect, they suggested getting help and support from hospital staff in early hours, and receiving support from social mental health teams in later hours. They also suggested to store disposable items such as stents, balloons, intra-uterine devices (IUDs), and pacemakers along with toothpaste, toothbrush, sanitary pad, mother-child packs as strategic items.

 Gil and McNeil dissected the level of outsourcing among the performing artists of a compassionate reaction framework to help those affected by normal calamities, and to help those affected by the outfitted strife in Colombia.^10^ Supply chains were codified in four fragments for investigation purposes: coordination operations supporting the Fiasco region (upstream), the catastrophe region (midstream), and the specific recipients (downstream and final mile dispersion as two separate sections). A number of third parties supported organizations and private temporary workers, through partaking within the supply chains as on-screen characters, and organizing nourishment help conveyance to recipients. These bunches were dissected to demonstrate contrasts and commonalties, and reach conclusions of common appropriateness.The study provided arrangement suggestions to extend the execution of future nourishment help dispersion operations in reaction to bigger scale calamities. Overall, this study suggested the delivery of warehousing and transportation equipment to third party logistics, the delivery of food aid kits to local authorities, and material handling to downstream transportation management.

 Thomas and Kopczak also conducted a descriptive study to synthesize data regarding the logistics employed in the mass disaster management.^12^ The study focused on the following aspects: the role and importance of media, management of donations, and coordination of humanitarian logistics. In the case herein analyzed, the humanitarian response was considered to be adequate, especially in the survivors’ care. Finally, in the study, an efficient coordination strategy was adopted, and the media was found with a vital role in requesting blood donations.

###  Methodological quality

 Overall, five out of the seven included studies were found to be with high quality (4 > points) in terms of the following items: type of study, description of control group, definition of exclusion criteria, definition of outcome, ethical approval, statistical analysis, sample size calculation, and description of patient group. The results of qualitative review are presented in detail in Figure 2.

## Discussion

 This study aimed to systematically explore the available logistic models for disasters management, and to compare systems of different countries regarding their human resources, infrastructure, storage, priority items, distribution, access system, coordination of organizations, and information communication with the media. In this respect, we identified seven studies that matched our inclusion criteria. These studies were mainly from Iran, the United States, and Indonesia, which are among the countries with high rates of natural disasters and high need to perform logistics.

 Each included model in this study focused mainly on a specific aspect of disaster management. For instance, one of the Indonesia’s latest and easily applicable models discussed the use of technology to sufficiently distribute the available resources, and to coordinate teams in disaster management. It has also suggested the application of smart government, and the developing of a smartphone app named e-logpal.^7^ There is a struggle in the governance and administration of disaster management equipment. The e-logpal application seems not yet to be ideal, and still requires enhancement, because it has been running for two years and still needs to be updated manually. Moreover, data update needs approval from each region and cannot be updated automatically. However, as we live in a world where everyone has access to smartphones and electronic connection devices, it seems to be one of the most useful strategies in natural disaster management.

 Overall, the present review showed that human resources in disaster management consists of a logistic team, an assessment team, and warehouse providers. It is also possible to use voluntaries, military services, and health care professionals to manage the initial phase of natural disasters regarding their medical issues and psychological effects.^13^ Also, the required items can be delivered by air and sea routes as alternative routes. In this respect, different algorithms can effectively solve the problem of infrastructures on small and large scales, such as the particle swarm optimization algorithm. Also, as previously discussed, each region can have an e-logpal account to sufficiently monitor its equipment and logistics data.

 Furthermore, essential prioritized items to be delivered may include basic medical equipment (including standard and custom rescue kits) for the treatment of trauma and acute illnesses, water (beverages and non-potable), non-renewable resources, food aid packages, toothpaste, and toothbrush, sanitary pads, mother-child packs, portable shelter, bed, blanket, generator, plywood, and nail.

 There are also many different opinions concerning distribution and access systems in disastrous conditions. One of included studies discussed and compared the UN Cluster Approach, which leaves warehousing and transportation to third-party logistics, with the Unit for Assistance and Reparation of Victims approach (UARIV). This approach consists of delivering food aid kits to local authorities and allowing them to handle supplies and manage local transportation.^10,14^ The essential parts of the models were utilizing all possible delivery routes (including air, land, and sea), setting up field hospitals, and technology and mobile apps to manage the situations effectively.

 To bargain with the challenges of large-scale calamities, we need to alter numerous fundamental presumptions that we usually use in conventional trade coordination. Depended on different occasions, we can move our center from issue deterioration to multi-task integration, from time for operation effectiveness to time for life sparing, from single decision-making specialist to multiparty specialist, from boundless asset accessibility to genuine asset deficiency, from vulnerability with arbitrariness to profound vulnerability without past information, and from idealize transportation foundation to harmed infrastructure.^15^

 Combining all logistic data from previously published studies enabled us to understand all possible and previously discussed issues in disaster management, to compare the available hypotheses, and to pick up the best ones to be ready for the management of disasters, effectively. However, there are also some limitations. For instance, we only included the articles published in English in our review. Therefore, many locally published articles, especially in countries with higher disaster rates, may not be included in the study. Also, we could not conduct numerical analyses on the previously published data of the articles, due to the heterogeneity of the models and algorithms. However, the results, methods, and strategies of previous studies were summarized and combined. It should be also kept in mind that each emergency is special with its unique characteristics: there’s no such thing as a uniform reaction for each incident.^16^ So, we hope that this article will pave the way for achieving more efficient disaster management, worldwide, and reduce the rate of disaster casualties.

## Conclusion

 This study systematically highlighted the crucial points that should be considered in managing natural disasters including human resources, infrastructure, storage, priority items, distribution, access system, coordination of organizations, information, and communication with the media. In this regard, we prepared a comprehensive comparison of possible models and logistics. It is also recommended to conduct further studies to summarize the results of numerous articles in languages other than English to have a better understanding of local strategies.

## Acknowledgements

 This study was part of a PhD Thesis supported by Iran University of medical science. (No: IUMS/SMMIS_99_2_37_18958). We also thank Editors and Reviewers of the Journal Health Promotion Perspectives for their valuable comments.

## Author Contributions

**Conceptualization:** Hesam Seyedin.

**Data curation: **Yousef Pashaei Asl.

 F**ormal Analysis: **Yousef Pashaei Asl

**Funding acquisition: **Hesam Seyedin.

**Investigation: **Javad Babaie, Mohsen Dowlati.

**Methodology: **Yousef Pashaei Asl.

**Project administration:** Hesam Seyedin.

**Resources: **Yousef Pashaei Asl

**Software: **Yousef Pashaei Asl, Mohsen Dowlati.

**Supervision: **Javad Babaie.

**Validation: **Hesam Seyedin.

**Visualization: **Mohsen Dowlati, Javad Babaie.

**Writing – original draft: **Hesam Seyedin, Yousef Pashaei Asl, Mohsen Dowlati, Javad Babaie.

## Funding

 No funding was available in this study.

## Ethical Approval

 Not applicable.

## Competing Interests

 All authors declare that no competing interests exist.
